# Predicting cholangiocarcinoma in primary sclerosing cholangitis: using artificial intelligence, clinical and laboratory data

**DOI:** 10.1186/s12876-023-02759-7

**Published:** 2023-04-19

**Authors:** Chang Hu, Ravishankar K. Iyer, Brian D. Juran, Bryan M. McCauley, Elizabeth J. Atkinson, John E. Eaton, Ahmad H. Ali, Konstantinos N. Lazaridis

**Affiliations:** 1 Department of Electrical and Computer Engineering, University of Illinois at Urbana-Champaign, Urbana-Champaign, IL 61801 USA; 2 Division of Gastroenterology and Hepatology, College of Medicine, Mayo Clinic, 200 First Street SW, Rochester, MN 55905 USA; 3 Division of Biomedical Statistics and Informatics, Mayo Clinic, Rochester, MN 55905 USA; 4 Division of Gastroenterology and Hepatology, University of Missouri School of Medicine, Columbia, MO 65212 USA

**Keywords:** Primary sclerosing cholangitis, Cholangiocarcinoma, Risk factors, Artificial intelligence, Bile acids

## Abstract

**Background:**

Primary sclerosing cholangitis (PSC) patients have a risk of developing cholangiocarcinoma (CCA). Establishing predictive models for CCA in PSC is important.

**Methods:**

In a large cohort of 1,459 PSC patients seen at Mayo Clinic (1993–2020), we quantified the impact of clinical/laboratory variables on CCA development using univariate and multivariate Cox models and predicted CCA using statistical and artificial intelligence (AI) approaches. We explored plasma bile acid (BA) levels’ predictive power of CCA (subset of 300 patients, BA cohort).

**Results:**

Eight significant risk factors (false discovery rate: 20%) were identified with univariate analysis; prolonged inflammatory bowel disease (IBD) was the most important one. IBD duration, PSC duration, and total bilirubin remained significant (*p* < 0.05) with multivariate analysis. Clinical/laboratory variables predicted CCA with cross-validated C-indexes of 0.68–0.71 at different time points of disease, significantly better compared to commonly used PSC risk scores. Lower chenodeoxycholic acid, higher conjugated fraction of lithocholic acid and hyodeoxycholic acid, and higher ratio of cholic acid to chenodeoxycholic acid were predictive of CCA. BAs predicted CCA with a cross-validated C-index of 0.66 (std: 0.11, BA cohort), similar to clinical/laboratory variables (C-index = 0.64, std: 0.11, BA cohort). Combining BAs with clinical/laboratory variables leads to the best average C-index of 0.67 (std: 0.13, BA cohort).

**Conclusions:**

In a large PSC cohort, we identified clinical and laboratory risk factors for CCA development and demonstrated the first AI based predictive models that performed significantly better than commonly used PSC risk scores. More predictive data modalities are needed for clinical adoption of these models.

**Supplementary Information:**

The online version contains supplementary material available at 10.1186/s12876-023-02759-7.

## Background

Primary sclerosing cholangitis (PSC) is an immune-mediated cholestatic liver disease characterized by inflammation and fibrosis of the bile ducts, often progressing to end-stage liver disease requiring liver transplantation (LT). Nearly 75%–80% of patients with PSC have comorbid inflammatory bowel disease (IBD) [1] and are at high risk for developing cholangiocarcinoma (CCA) [2]. The lifetime risk of CCA in PSC patients has been reported to be 7%–13% [3]. While somewhat treatable when detected early, CCA remains a significant cause of mortality in PSC patients, due to lack of clinically useful prediction tools [4]. Ability to predict development of CCA in PSC patients could lead to better surveillance programs capable of identifying CCA at a curable stage resulting in improved outcomes.

Prior studies have identified risk factors for CCA in PSC patients including advanced age, male sex, and prolonged duration of IBD [5–9]. However, many of these studies suffered from small sample sizes, resulting in highly uncertain estimates of the effect sizes of the risk factors. Bile acids (BAs) have been proposed as important chemicals implicated in the development and pathogenesis of PSC and can be measured using inexpensive and noninvasive clinical assays [10].

Personalized risk prediction models, such as the Primary Sclerosing Cholangitis Risk Estimate Tool (PREsTo) [11], are currently used as clinical decision tools for estimating survival rates and hepatic decompensation events in PSC. However, large sample sizes and a significant number of events are usually required to train the models reliably, which has hindered development of such models to predict CCA in PSC.

In this study, we leverage one of the largest single-center well-phenotyped PSC cohorts to better understand the clinical/laboratory and plasma BA signatures and identify risk factors for CCA development in PSC. Using a rigorous analytical approach employing statistical and artificial intelligence (AI) approaches, we aim to narrow the gap in individualized treatment of PSC through personalized CCA risk prediction models. We expect our approach to be adaptable for future studies focusing on comprehensive multi-omics profiling of PSC patients, with the goal of providing better clinical management tools for PSC through omics based individual risk-informed surveillance programs.

## Methods

### Patient population

The cohort of patients with PSC in this study are enrolled in the PSC Scientific Community Resource [12]. A detailed explanation and mechanisms of patients’ enrollment, consenting, chart review, data collection, questionnaires, and biospecimens have been recently described [12]. Briefly, patients with PSC who receive their medical care at the three main Mayo Clinic sites (Minnesota, Florida, and Arizona) and in the broader Mayo Clinic Health System were identified by manual chart review and invited in person or by mail to participate in our studies. All available medical charts (electronic and paper) were comprehensively reviewed by two experienced hepatologists. To be enrolled, patients must meet the following established diagnostic criteria for PSC according to the American Association for the Study of Liver Diseases guidelines [13]: (a) biochemical evidence of chronic cholestasis (≥ 6 months); (b) cholangiographic evidence of multifocal strictures and segmental dilatations in the bile ducts and/or histological features consistent with PSC; and (c) exclusion of secondary causes of sclerosing cholangitis. For each patient, demographics; clinical data relevant to PSC and IBD; laboratory; cholangiographic; histological; and endpoints’ data were extracted manually from patients’ charts. For the purpose of this study, charts of patients with PSC who developed CCA were re-reviewed by one of the hepatologists, and the following data regarding CCA were extracted: (a) date of CCA diagnosis; (b) cytology (negative; abnormal; atypical; suspicious; or positive for adenocarcinoma); (c) polysomy on fluorescence in situ hybridization (FISH); (d) serum carbohydrate antigen 19–9 (CA 19–9) closest to the date of diagnosis of CCA; (e) type of CCA (malignant-appearing stricture; mass); (f) histopathology (benign; reactive; low-grade dysplasia; high-grade dysplasia; or adenocarcinoma); (g) type of treatment of CCA, if any (partial hepatectomy; liver transplantation; en bloc resection of the bile ducts; or systemic chemotherapy); and (h) evidence of residual CCA in the explant after liver transplantation.

CCA was diagnosed by: (a) imaging/cholangiographic findings characteristic of CCA with positive cytology or histopathology; (b) malignant-appearing strictures with FISH polysomy plus suspicious cytology; (c) malignant-appearing strictures with FISH polysomy plus elevated serum CA 19–9; (d) malignant-appearing strictures with FISH polysomy; and (e) malignant-appearing strictures with persistently elevated serum CA 19–9 [14–16]. Occurrences of gallbladder cancer (GBC), hepatocellular carcinoma (HCC), LT, and death from causes other than PSC were recorded and considered competing events. GBC and HCC were diagnosed according to published criteria [16–18]. This study was approved by the Mayo Clinic's Institutional Review Board. All participating subjects gave informed consent and thus, the study follows the ethical standards laid down in the 1964 Declaration of Helsinki and its later amendments.

### Data collection and preprocessing

Clinical variables and laboratory parameters were abstracted from the electronic medical record (EMR) of PSC patients and used in analysis of the baseline cohort. A subset of these patients had plasma BA data available from a previous study [10]. This cohort, defined as the BA cohort for the purpose of this study, was used to evaluate the potential of BAs to improve prediction of CCA (Fig. S1). Cinical variables included sex, date of birth, date of PSC diagnosis, date of IBD diagnosis (if applicable), event dates (diagnosis of CCA, GBC, HCC, LT, and death), date of last clinical encounter, and disease severity of PSC at the time of PSC diagnosis assessed by the Model for End-stage Liver Disease (MELD) score [19], Mayo PSC Risk Score [20], and PREsTo score [11]. Diagnosis dates of PSC, IBD, CCA, GBC, and HCC were shifted to 30 days prior to the documented dates to accommodate testing performed to establish the diagnoses. The first available laboratory test results in the Mayo EMR following PSC diagnosis and prior to any of the outcomes (i.e., CCA, GBC, HCC, LT, and death) were extracted and used as baseline measurements for prediction. These laboratory tests included albumin, alkaline phosphatase (ALK), alanine aminotransferase (ALT), aspartate aminotransferase (AST), bilirubin (total and direct), CA 19–9, complete blood count (hemoglobin, leukocytes, and platelet count), immunoglobulin G (total IgG and IgG4), international normalized ratio (INR), sodium, and creatinine. Missing laboratory parameters were imputed first using the closest measurement of the same laboratory parameter within the past one year or the next seven days after baseline. Two separate strategies were adopted to impute the remaining missing laboratory parameters: (i) for risk factor identification, we adopted the strategy of multiple imputation to get unbiased *p*-value estimates from the pooled results using the predictive mean matching method in the Multivariate Imputation by Chained Equations (MICE) package [21] (version 3.14.0); and (ii) for predictive models construction, we integrated missing data imputation into the cross-validation process. Specifically, we used the training data to learn to impute each feature from all other features with the scikit-learn [22] (version 0.24.2) package.

Plasma primary and secondary BAs data (described in Mousa et al. [10]) available for a subset of the patients included: CA, CDCA, DCA, LCA, UDCA, HDCA; and their taurine conjugated forms: TCA, TCDCA, TDCA, TLCA, TUDCA, THDCA; and their glycine conjugated forms: GCA, GCDCA, GDCA, GLCA, GUDCA, and GHDCA (see list of abbreviations). The total BA concentration was calculated by summing the concentrations of all evaluated BAs. Total concentration of BA “families” were calculated by summing the unconjugated and conjugated forms (e.g., Total CA = CA + GCA + TCA). Conjugated fraction was calculated as the sum of the conjugated forms divided by the total (e.g., ConFrac CA = [GCA + TCA]/[CA + GCA + TCA]). The G:T conjugation ratios were calculated by dividing the glycine-conjugated form by the taurine-conjugated form (e.g., GTratio CA = GCA/TCA). GTratio HDCA was excluded due to the high percentage of zero values (undetectable) in THDCA concentrations. Ratios of CA:CDCA, CA:DCA, and CDCA:(LCA + HDCA + UDCA) were calculated using the “total BA” family concentrations. For fractions and ratios, values were set to blank if the denominator was equal to zero. Patients in whom BAs measurements were performed after developing any of the outcomes (i.e., CCA, GBC, HCC, LT, and death) were excluded. To synchronize the measurement time of the BAs and the laboratory parameters, we queried the EMR and abstracted laboratory test results collected closest to and within one year of the BAs measurement. Patients without laboratory data within this interval were excluded. Hence, the BA cohort was a “time-shifted” subset of the baseline cohort wherein laboratory parameters close to the BAs measurement time were used instead of the baseline values.

### Incidence of CCA

We analyzed the incidence of CCA in patients with PSC treating GBC, HCC, LT, and death from causes other than PSC as competing events. Patients who did not develop CCA or any of the competing events were censored at the last known clinical encounter. We used the mstate package [23] to generate the cumulative incidence functions (CIF) for the probability of developing CCA in light of the competing events.

### Identifying risk factors

We used Cox proportional hazards models (CoxPH) [24] to identify risk factors for the development of CCA. Censoring was made at the time of GBC, HCC, LT, death, or the last clinical encounter, whichever occurred first. Patients without an IBD diagnosis were considered to have an IBD duration of 0 year. To make the hazard ratios (HRs) estimated from the CoxPH models more straightforward to interpret, we categorized age into nine bins of 10-year intervals and kept year as the unit for PSC duration and IBD duration at baseline. We normalized MELD score, Mayo PSC Risk Score, PREsTo score, hemoglobin, and sodium by dividing the actual values by their interquartile range (IQR). Continuous laboratory parameters and BAs with zero value (undetectable) were replaced with half of the smallest nonzero values. Laboratory parameters and BAs apart from hemoglobin and sodium were log-transformed (base 10) because they were highly right-skewed.

We first constructed univariate CoxPH models for each baseline clinical variable and laboratory parameter and reported their *p*-values and HRs with the 95% confidence interval (CI). The Benjamini–Hochberg procedure [25] was performed to control the false discovery rate (FDR) to be below 20%. Using baseline factors that passed the FDR threshold, we constructed a multivariate CoxPH model to estimate the combined effect of the baseline factors and assessed each factor’s influence in the presence of other features. We did not consider the composite scores (Mayo PSC Risk Score, PREsTo score, and MELD score) in the multivariate model because they are calculated from laboratory parameters and will obscure the HR interpretation. Additionally, we excluded direct bilirubin because it was found to be highly correlated with total bilirubin (Pearson correlation = 0.98). A similar approach was used in analysis of the BA cohort.

### Predictive modeling

We first constructed a set of models using baseline clinical variables and laboratory parameters of the baseline cohort. Censoring was again made at the time of GBC, HCC, LT, death, or the last clinical encounter, whichever occurred first. Patients without an IBD diagnosis were considered to have an IBD duration of 0 year. We similarly excluded the composite scores and direct bilirubin. We excluded total IgG and IgG4 for their high missingness. We applied Yeo-Johnson power transformation [26] to the highly right-skewed features (same as the log-transformed variables in risk factor identification). We then standardized all the continuous features, including the power-transformed features.

Three models were used in predicting CCA: (i) the abovementioned multivariate CoxPH model with regularization term(s); (ii) Random Survival Forest (RSF) [27], and (iii) Gradient Boosting Survival Analysis (GBSA) [28]. CoxPH assumes that the HR of two subjects is constant over time and that the population of interest shares a common baseline hazard function. We used the nonparametric Breslow's method [29] to estimate the baseline hazard. We added l2 and l1 regularization terms to reduce the chance of overfitting and encourage the selection of fewer features. We set the regularization parameters to 0.005 for both terms. Both RSF and GBSA are tree-based ensemble AI methods that automatically handle the nonlinear relationship between features and outcomes. RSF is one of the most popular learning-based AI alternatives to CoxPH for survival analysis [30], while gradient boosting methods have been widely used and proven successful in prediction competitions [31] and medical applications [11]. RSF leverages bootstrapping to construct multiple survival trees and average their results for a robust prediction. We chose the log-rank statistic as the splitting rule for building the survival trees. We used 100 trees for stable performance according to the weak law of large numbers. GBSA iteratively learns an ensemble of decision trees that maximize the partial log-likelihood of the observed survival outcomes. We set the number of trees to be the default value of the software, which was also 100. For both RSF and GBSA, we set each tree's maximum depth to 3 to allow nonlinear 3-way interactions among the features when making predictions. We required a minimum of 30 patients at each leaf node for reliable estimation. We implemented all three methods in Python using the scikit-survival package [32] (version 0.16.0).

We evaluated the predictive performance using the concordance index (C-index). C-index values range from 0 to 1. A high C-Index indicates the model correctly predicts higher risk for patients who developed CCA in shorter times. We calculated the mean and standard deviation of the test set C-index from a 20-fold Monte Carlo cross-validation with 80%–20% train–test split. Specifically, we randomly split the dataset into a training set (80%) and a test set (20%) and repeated this process 20 times. The results were calculated by taking the mean and standard deviation of the test set C-index across all 20 splits. All models shared the same train–test splits to ensure a fair comparison.

We calculated the permutation feature importance by measuring decrease in C-index of each model when the values of a feature were randomly permuted (across test set patients). For each cross-validation fold, the permutation was repeated three times, and the mean decrease in the test set C-index was evaluated. To assess how well the model performs at different time points in the disease course, we also evaluated the models using clinical variables and laboratory parameters collected at 2 and 5 years post PSC diagnosis. Time related clinical variables such as age and disease duration were updated according to the status at the evaluated time points.

For the BA cohort, we constructed a second set of models using plasma BAs and/or clinical variables and laboratory parameters. Composite BA variables with no blank values were also included. We employed the same methodology as above, except due to the smaller number of patients and CCA occurrences in the BA cohort, we adopted a more balanced 70%–30% train–test split and performed recursive feature elimination on the training set to select the three most important BAs associated with CCA. In CoxPH, since feature selection was explicitly performed, we dropped the l1 regularization term and used only an l2 regularization term with 0.01 as the parameter. We kept the choices of other hyperparameters consistent with the models for the baseline cohort. The models were then trained with the selected BAs, and C-index was calculated on the test set. To compare the predictive power of BAs, clinical variables, and laboratory parameters, we repeated the abovementioned procedure replacing BAs with clinical/laboratory variables. Furthermore, we trained models combining the three selected BAs and the three selected clinical/laboratory variables from the corresponding cross-validation fold. Feature importance was again measured with three permutations.

## Results

### Baseline cohort

A total of 1,459 PSC patients were included in the baseline cohort, and their characteristics are summarized in Table 1. Median age at baseline was 44.2 years (IQR: 32.7–55.2) and 64.2% of the patients were male. Median time from PSC diagnosis to baseline (i.e., the first available laboratory test following PSC diagnosis) was 0.56 years (IQR: 0.16–3.47). One thousand thirty-five patients (70.9%) had received a diagnosis of IBD at baseline, with a median IBD duration of 8.45 years (IQR: 2.3–19.51). In the baseline cohort, 125 cases of CCA (8.6%), 15 GBC (1.0%), and 32 HCC (2.2%) were recorded. Four patients had both CCA and GBC, and two had both CCA and HCC. The median time from PSC diagnosis until the last clinical encounter was 10.5 years (IQR: 5.2–17.8). As shown in Fig. S2, the cumulative incidence of CCA grew linearly with time, representing a constant incidence rate. The cumulative incidence of CCA was found to be 2.2%, 5.3%, 8.4%, and 15.9% at 2, 5, 10, and 20 years from the time of PSC diagnosis, respectively.

**Table 1:** Summary characteristics of the baseline cohort

Feature	N	Summary
Age, years	1,459	44.19 (32.73–55.18)
Male sex	1,459	64.2%
PSC duration, years	1,459	0.56 (0.16–3.47)
IBD diagnosis	1,459	70.9%
IBD duration, years	1,035	8.65 (2.3–19.51)
MELD Score	1,081	5.78 (3.11–8.86)
Mayo PSC Risk Score	1,296	0.17 (-0.52–1.07)
PREsTo Score	1,039	0.04 (0.03–0.1)
ALK x ULN	1,409	2.21 (1.22–3.84)
AST, U/L	1,398	62 (36–105)
ALT, U/L	1,092	78 (43.75–136)
Total bilirubin, mg/dL	1,394	0.9 (0.6–1.7)
Direct bilirubin, mg/dL	1,131	0.3 (0.2–0.8)
INR	1,099	1 (0.9–1.1)
Albumin, g/dL	1,270	4 (3.6–4.3)
Sodium, mmol/L	1,004	140 (138–141)
Creatinine, mg/dL	1,321	0.9 (0.8–1.1)
Hemoglobin, g/dL	1,240	13.5 (12.2–14.7)
White Blood Cells, 10^9^/L	1,209	6.5 (5.2–8.4)
Platelets, 10^9^/L	1,362	247.5 (193–318)
IgG4, mg/dL	364	37.35 (18.5–81.58)
Total IgG, mg/dL	126	1305 (1085–1745)
CA 19–9, U/mL	902	17.65 (9–42)

Continuous features expressed as median (IQR)

Binary features expressed as percentage

### Diagnosis of CCA

Of the 118 PSC patients who developed CCA prior to any other outcomes, the diagnosis of CCA was established/confirmed pretreatment and/or posttreatment histopathologically and/or cytologically in 78.8% (93/118). To elaborate, pre-CCA treatment cytology positive for adenocarcinoma was identified in 52 patients and histopathology positive for adenocarcinoma in 20 patients. Further, histopathology on liver explant positive for adenocarcinoma was identified in 8 patients, histopathology on partial hepatectomy specimens positive for adenocarcinoma in 6 patients, fine needle aspiration of liver/metastatic mass positive for adenocarcinoma in 6 patients, and on en bloc resection of the bile ducts in 1 patient. Of the 25 patients without tissue diagnosis, 8 had clear evidence of CCA by a visible mass on abdominal cross-sectional imaging, 6 patients had malignant-appearing stricture with suspicious cytology and polysomy on FISH, 6 patients had malignant-appearing strictures with negative cytology but polysomy on FISH, and 5 patients had malignant-appearing strictures and elevated serum CA 19–9.

### Risk factor Identification

The HRs of the baseline clinical variables and laboratory parameters from the univariate CoxPH models are summarized in Table 2. Longer IBD duration, longer PSC duration, IBD diagnosis, higher total bilirubin, higher Mayo PSC Risk Score, higher CA 19–9, higher direct bilirubin, and older age have been found to be independent predictors of CCA development in the descending order of significance (FDR < 20%). Lower sodium, higher white blood cell count, higher PREsTo score, higher MELD score, and male sex all had q-values of 0.221, slightly above the 20% FDR threshold. The multivariate CoxPH model using baseline features that passed the FDR threshold of 20% in the univariate models is summarized in Table 3. Longer IBD duration, longer PSC duration, and higher total bilirubin were statistically significantly predictive of CCA (*p* < 0.05).

**Table 2:** Univariate associations with development of CCA in the baseline cohort

Variable	N	Hazard Ratio (95% CI)	*p*-value	q-value
IBD duration, years	1,459	1.05 (1.03–1.06)	1e-11	3e-10
PSC duration, years	1,459	1.08 (1.05–1.12)	1e-07	1e-06
IBD diagnosis	1,459	2.34 (1.43–3.83)	7e-04	0.005
Total bilirubin, (log 10)	1,394	2.19 (1.37–3.51)	0.001	0.006
Mayo PSC Risk Score, (IQR)	1,296	1.61 (1.19–2.16)	0.002	0.008
CA 19–9, (log 10)	901	1.76 (1.23–2.54)	0.002	0.008
Direct bilirubin, (log 10)	1,118	1.8 (1.23–2.65)	0.003	0.008
Age, (per 10 years)	1,459	1.21 (1.06–1.38)	0.004	0.011
Sodium, (IQR)	1,004	0.89 (0.77–1.02)	0.087	0.221
White Blood Cells, (log 10)	1,209	2.62 (0.84–8.17)	0.097	0.221
PREsTo Score, (IQR)	1,039	1.07 (0.98–1.16)	0.109	0.221
MELD Score, (IQR)	1,081	1.24 (0.95–1.62)	0.117	0.221
Male sex	1,459	1.36 (0.92–2.02)	0.125	0.221
INR, (log 10)	1,099	0.08 (0–3.04)	0.171	0.281
ALK, (log 10)	1,409	1.22 (0.69–2.14)	0.497	0.750
AST, (log 10)	1,398	1.21 (0.68–2.13)	0.522	0.750
Hemoglobin, (IQR)	1,240	0.93 (0.72–1.21)	0.597	0.807
Total IgG, (log 10)	126	2.23 (0.04–137.08)	0.702	0.822
Creatinine, (log 10)	1,321	0.72 (0.13–4.05)	0.709	0.822
ALT, (log 10)	1,092	1.11 (0.62–2)	0.715	0.822
Platelets, (log 10)	1,362	0.89 (0.34–2.34)	0.818	0.868
Albumin, (log 10)	1,270	1.45 (0.05–42.63)	0.830	0.868
IgG4, (log 10)	364	1.03 (0.53–2.03)	0.923	0.923

q-values are the Benjamini–Hochberg procedure adjusted *p*-values

**Table 3:** Multivariate associations with development of CCA in the baseline cohort

Variable	Hazard Ratio (95% CI)	*p*-value
IBD duration, years	1.04 (1.02–1.05)	2e-04
PSC duration, years	1.05 (1.02–1.08)	0.003
Total bilirubin, (log 10)	2.05 (1.23–3.41)	0.006
CA 19–9, (log 10)	1.4 (0.97–2.04)	0.071
IBD diagnosis	1.57 (0.87–2.83)	0.132
Age, (per 10 years)	1.06 (0.9–1.24)	0.501

Only baseline features with estimated FDR (q-values) less than 20% from the univariate models were considered

### Predictive modeling

The test C-index values of different models in predicting CCA-free survival for the baseline cohort using clinical variables and laboratory parameters are shown in Table 4. At baseline, CoxPH had the highest average C-index (0.69 (std: 0.06)) of the three models, followed by RSF (C-index = 0.68, std: 0.06). All three predictive models performed significantly better compared to the commonly used PSC scores, including the Mayo PSC Risk Score, the MELD score, and the PREsTo score in predicting CCA development (*p* < 0.005 comparing any of the predictive models with any of the risk scores). We evaluated performance of the models at future time points, 2 and 5 years post PSC diagnosis. The C-index values of each model at 2 and 5 years post PSC diagnosis remained unchanged compared to baseline.

**Table 4:** CCA predictive modeling C-Index in the baseline cohort

Test Scenario	Baseline	2 years post DX	5 years post DX
CoxPH	0.69 ± 0.06	0.68 ± 0.06	0.67 ± 0.11
RSF	0.68 ± 0.06	0.67 ± 0.06	0.71 ± 0.09
GBSA	0.65 ± 0.06	0.64 ± 0.07	0.66 ± 0.09
Mayo PSC Risk Score	0.57 ± 0.08		
MELD Score	0.53 ± 0.08		
PREsTo Score	0.57 ± 0.07		

• Results shown in mean ± std estimated from 20-fold Monte Carlo cross-validation with 80%–20% train–test split

• Row 1–3 show models trained using baseline clinical variables and laboratory parameters

• Row 4–6 show C-index of commonly used risk scores

• Pairwise comparison between row 1–3 and row 4–6 show that the predictive models are significantly better than all individual risk scores (*p*-value < 0.05)

• The three columns show test C-index using variables collected at: baseline, 2 years post PSC diagnosis (DX), and 5 years post DX

**Fig. 1:**
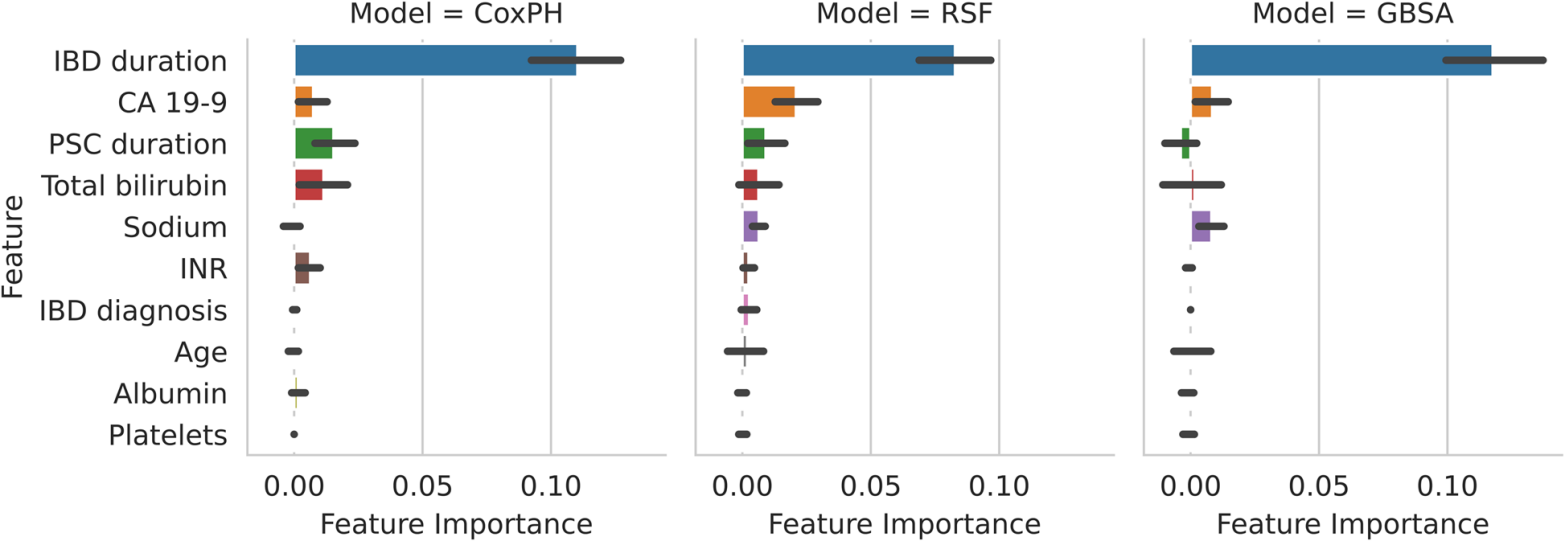
Permutation feature importance of the baseline clinical variables and laboratory parameters. The height of each rectangle shows the average importance (across cross-validation folds) of that feature. The error bars represent the 95% confidence interval. Features were ranked in a descending order according to the sum of their feature importance across models and cross-validation folds. Only the top 10 features are shown. Abbreviations: CA 19–9, carbohydrate antigen 19–9; CoxPH, Cox Proportional Hazards; RSF, Random Survival Forest; GSBA, Gradient Boosting Survival Analysis; IBD, inflammatory bowel disease; INR, international normalized ratio; PSC, primary sclerosing cholangitis

The permutation feature importance for the baseline clinical variables and laboratory parameters is shown in Fig. 1. IBD duration had the greatest feature importance for all three models, with an average of 0.11 (CoxPH), 0.08 (RSF), and 0.12 (GBSA) decrease in C-index when replaced with randomly permuted values. Among the remaining features, CA 19–9 level, PSC duration, total bilirubin, and sodium had the largest sum of feature importance across models.

### Impact of BAs on predicting CCA

Of the 1,459 PSC patients, 300 had BA data and constituted the BA cohort. The median time from PSC diagnosis until the last clinical encounter in this cohort was 9.6 years (IQR: 5.6–17.1), and during follow-up, 21 (7.0%) of the 300 patients were diagnosed with CCA. BA values, clinical variables and laboratory parameters of the BA cohort are provided in Table S1. Univariate analysis of BAs’ ability to predict CCA is provided in Table 5. Among individual BAs, only CDCA was statistically associated with CCA after controlling for FDR, with increased levels appearing to be protective against CCA. Conjugated fraction of LCA and HDCA as well as the ratio of CA:CDCA were also found to be predictive, with increased values associated with higher risk of CCA. Notably, increased conjugated fractions of total BA, CA, and CDCA were also nominally associated with increased risk of CCA (*p* < 0.05), although they were not statistically significant when controlling for FDR.

**Table 5:** Univariate associations with development of CCA in the bile acid cohort

Variable	N	Hazard Ratio (95% CI)	*p*-value	q-value
CDCA	300	0.34 (0.16–0.72)	0.004	0.18
ConFrac LCA	217	37.44 (2.14–656.64)	0.013	0.18
CA:CDCA	300	1.48 (1.08–2.03)	0.015	0.18
ConFrac HDCA	178	6.66 (1.4–31.76)	0.017	0.18
ConFrac all BA	300	95.31 (1.75–5180.88)	0.025	0.21
ConFrac CA	300	460.41 (1.48–143,616.99)	0.036	0.23
ConFrac CDCA	300	273.58 (1.17–64,085.63)	0.044	0.23
CA	300	0.45 (0.2–0.99)	0.048	0.23
GTratio DCA	223	0.88 (0.77–1)	0.055	0.23
UDCA	300	0.66 (0.44–1.01)	0.056	0.23
GTratio all BA	299	0.96 (0.92–1)	0.061	0.23
TCA	300	1.47 (0.96–2.27)	0.079	0.27
GHDCA	300	2.36 (0.87–6.42)	0.094	0.28
GTratio CDCA	299	0.93 (0.85–1.01)	0.095	0.28
GTratio UDCA	247	0.98 (0.96–1)	0.109	0.30
Total UDCA	300	0.74 (0.51–1.08)	0.124	0.32
GUDCA	300	0.75 (0.52–1.09)	0.135	0.32
TDCA	300	1.46 (0.89–2.41)	0.138	0.32
GTratio LCA	130	0.82 (0.63–1.07)	0.151	0.33
GTratio CA	295	0.93 (0.84–1.03)	0.163	0.33
GCA	300	1.47 (0.84–2.55)	0.177	0.35
TLCA	300	1.66 (0.75–3.72)	0.214	0.40
TCDCA	300	1.38 (0.82–2.33)	0.232	0.41
Total CA	300	1.4 (0.79–2.48)	0.246	0.42
HDCA	300	0.53 (0.17–1.67)	0.279	0.46
ConFrac DCA	261	2.58 (0.39–16.94)	0.323	0.51
LCA	300	0.68 (0.29–1.56)	0.359	0.55
DCA	300	0.8 (0.49–1.31)	0.384	0.56
Total HDCA	300	0.75 (0.3–1.9)	0.549	0.76
ConFrac UDCA	294	2.18 (0.16–29.76)	0.559	0.76
TUDCA	300	0.88 (0.54–1.44)	0.622	0.82
GDCA	300	1.12 (0.71–1.76)	0.637	0.82
Total DCA	300	1.09 (0.71–1.68)	0.682	0.85
GLCA	300	1.1 (0.58–2.11)	0.771	0.93
GCDCA	300	1.09 (0.51–2.34)	0.816	0.94
Total Bile Acids	300	0.93 (0.45–1.94)	0.850	0.94
CA:DCA	261	1 (1–1)	0.864	0.94
Total CDCA	300	1.06 (0.49–2.29)	0.874	0.94
Total LCA	300	1.03 (0.58–1.84)	0.920	0.97
CDCA:(LCA + HDCA + UDCA)	294	1 (1–1)	0.972	1.00
THDCA	300	0 (0–Inf)	0.997	1.00

All bile acids except the ratios were log-transformed (base 10)

*q*-values are the Benjamini–Hochberg procedure adjusted *p*-values

**Fig. 2:**
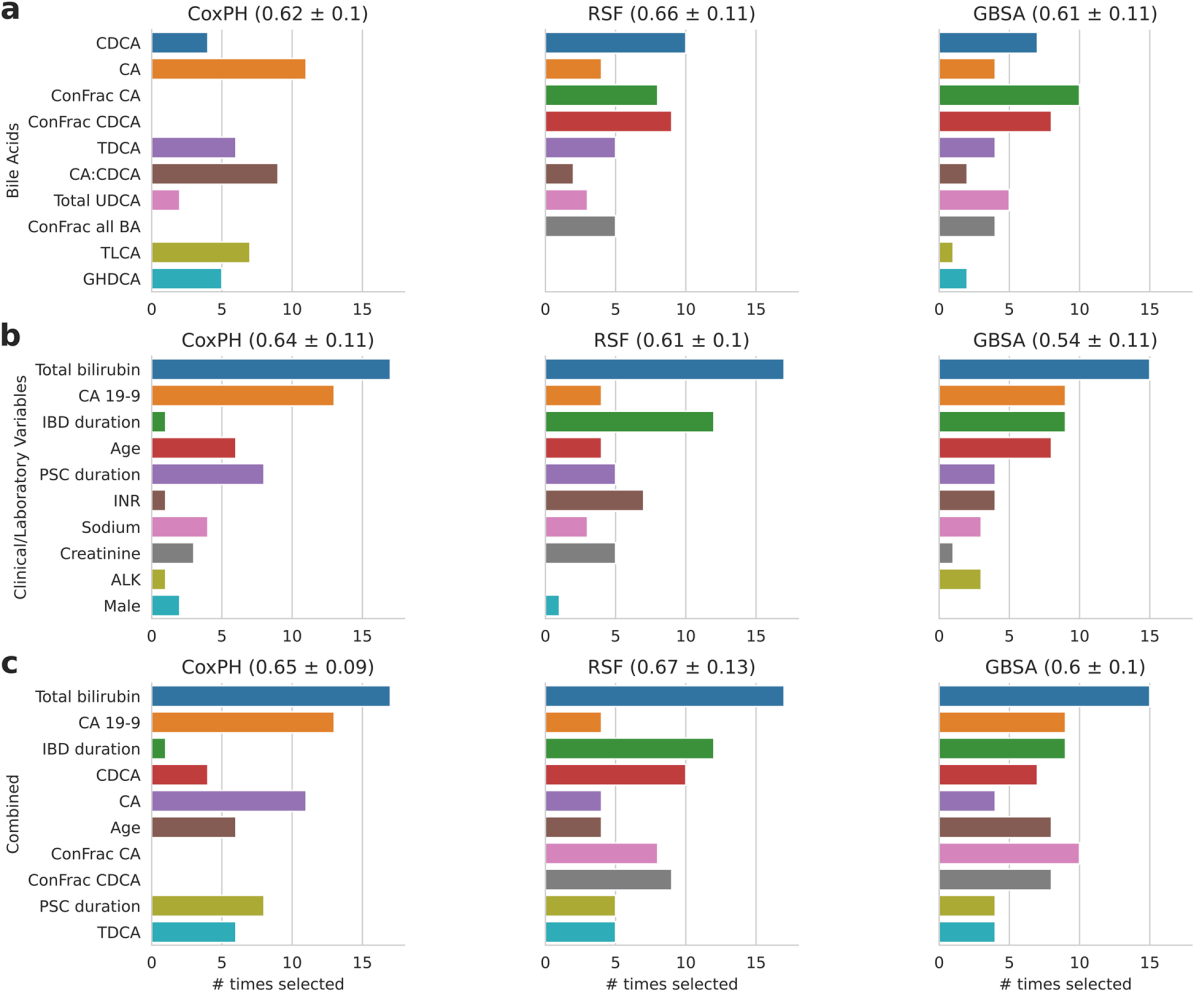
Most frequently selected features for the bile acid cohort with predictive modeling C-index shown in mean ± std estimated from 20-fold Monte Carlo cross-validation. The recursive feature elimination process selected three most important features in each of the 20 cross-validation folds. The height of each rectangle shows the number of times a feature was selected out of the 20 folds. Features were ranked in a descending order according to the sum of their selected times across models and cross-validation folds. Only the top 10 features are shown. Note that the feature selection process was only performed for **a** only bile acids and for **b** only clinical variables and laboratory parameters, and the selected features from **a** and **b** were combined to train the models in (**c**). Abbreviations: CA 19–9, carbohydrate antigen 19–9; CoxPH, Cox Proportional Hazards; RSF, Random Survival Forest; GSBA, Gradient Boosting Survival Analysis; IBD, inflammatory bowel disease; INR, international normalized ratio; PSC, primary sclerosing cholangitis. For a complete list of abbreviations of the bile acids please see abbreviations

We evaluated the predictive power of BAs compared to clinical variables and laboratory parameters on the BA cohort, with the most frequently selected features shown in Fig. 2. When only BAs were included in the modeling, RSF had the best average C-index of 0.66 (std: 011), with CDCA being the most frequently selected feature from the recursive feature elimination process, followed by conjugated fraction of CDCA and conjugated fraction of CA (Fig. 2a). While the GBSA model did not perform as well (average C-index of 0.61, std: 0.11), feature importance was similar to that of RSF. When only clinical variables and laboratory parameters were included in the modeling, CoxPH performed best, with an average C-index of 0.64 (std: 0.11) (Fig. 2b). The gain in performance using BA variables compared to clinical variables and laboratory parameters was significant for GBSA (*p* = 0.036) and marginally significant for RSF (*p* = 0.054). Whereas, the loss in performance using BA variables for CoxPH model was not significant (*p* = 0.572). When selected clinical variables, laboratory parameters, and BAs were combined, the RSF and GBSA models had improved performance compared to when using only the clinical/laboratory variables alone (*p* < 0.01) (Fig. 2c). Performance when combining all variables was comparable to using BAs alone.

## Discussion

In this study, we report that longer history of IBD and PSC as well as higher bilirubin and CA 19–9 were found to be the most important predictors of CCA in PSC patients. We showed that clinical variables and laboratory parameters predicted CCA significantly better than the commonly used risk scores. The results were generalizable over the course of PSC, showing similar performance at 2- and 5- years post PSC diagnosis. By studying a subset of patients, we found that BAs marginally improved CCA prediction beyond clinical variables and routine laboratory parameters.

CCA is one of the most common causes of morbidity and mortality in patients with PSC. Accurate biomarkers with high sensitivity and specificity for prediction of CCA in PSC are lacking. Worsening of liver biochemistry or cholestasis, and onset of symptoms such as abdominal pain and jaundice should raise the suspicion for CCA. However, many patients with PSC complicated by CCA are asymptomatic. Recently updated guidelines from the American Association for the Study of Liver Diseases recommend routine screening for CCA in patients with PSC by cross sectional imaging with or without CA 19–9 [33]. However, the imaging modality and the optimal cutoff CA 19–9 to be used for screening are stil a subject of debate [33]. Thus, identification of markers and strategies to build predictive models of CCA represents a significant area of unmet clinical need in PSC.

Our analysis showed that longer IBD duration was the most significant risk factor for CCA at baseline, which is in keeping with a previous report [7]. It is beyond the scope of this paper to further examine the link between IBD and CCA carcinogenesis, but we hypothesize that the prolonged bowel wall injury results in interruption of the intestinal barrier, which in turn leads to increased exposure of the biliary tree to tumor-promoting substances such as bacterial products/toxins and toxic BAs [34]. PSC duration at baseline was also a significant risk for CCA, which is not surprising, and highlights the ongoing risk of CCA in these patients.

CA 19–9 is a well-known serum tumor marker that has been found to be overexpressed by epithelial tumors of the gastrointestinal tract, such as pancreatic and biliary cancers [35]. In many medical centers, CA 19–9 is used as a screening marker for CCA in patients with PSC. The reported sensitivity and specificity of CA 19–9 for detecting CCA have varied, ranging between 50%–90% and 54%–98%, respectively [36]. While CA 19–9 has been reported to be a useful marker for predicting CCA in patients with PSC [37–42], there are some limitations, including its elevation in some non-CCA conditions such as smoking as well as in patients with benign biliary obstruction and ascending cholangitis [43]. Our univariate analysis showed that a tenfold increase in CA 19–9 level was associated with a 1.8-fold increase in the hazard of developing CCA, lending strength to the proposed value of CA 19–9 in predicting CCA in patients with PSC. Previous studies have reported conflicting findings regarding the utility of bilirubin in prediction of CCA in PSC patients. Burak et al. reported a univariate CoxPH model in which serum bilirubin was not found to be a significant risk factor for CCA development in PSC [8]. However, we found bilirubin to be statistically significant in both univariate and multivariate models, which is in keeping with multiple previous reports [41, 44, 45]. The relationship between bilirubin and CCA development is not clear, but we speculate that the serum bilirubin concentration rises in response to formation of biliary strictures which could lead to future development of CCA.

Although studies have identified risk factors for CCA in patients with PSC, individualized predictive models that can estimate the probability of CCA-free survival are lacking. To the best of our knowledge, we constructed the first individualized predictive models for predicting CCA-free survival in PSC. Although higher C-index values are desired, we believe our work represents a critical addition to the existing literature since our cohort is one of the largest single-center populations with well-documented PSC. Our models predicted CCA in PSC significantly better than the commonly used risk scores, including the Mayo PSC Risk Score, the MELD score, and the PREsTo score. While the Mayo PSC Risk Score and the MELD score used death as endpoints and the PREsTo used hepatic decompensation (ascites, variceal hemorrhage, or encephalopathy) as endpoint, they do not consider risk factors for CCA, such as IBD duration or CA19-9, which were shown to be the most important predictors in our individualized models. Data from our study substantiate the notion that CCA in PSC presents a complex interaction of clinical, biochemical, genetic, and environmental factors that it might not be possible to identify only using the routinely obtained clinical and laboratory variables.

Part of the putative complex interaction might be explained by the BAs. We hence examined plasma BA data as an additional data modality for the prediction of CCA development in PSC. Our results showed that, in the BA cohort, BAs predicted CCA with a C-index of 0.66. To put this number in context, in the same BA cohort, clinical variables and laboratory parameters predicted CCA with a C-index of 0.64, slightly worse than the BAs-based prediction. Combining selected BAs, clinical variables and laboratory parameters resulted in the best predictive performance with a C-index of 0.67. It is worth noting that the relatively small number of CCA cases available for training (15 CCA cases on average) hindered the models’ abilities to accurately learn the relationships between predictors and CCA. Furthermore, we only selected three features (six features in the combined scenario) to reduce overfitting. Since our preliminary results for the BA cohort suggest that BAs improved CCA prediction beyond clinical variables and routine laboratory parameters, it would be valuable to study the predictability of BAs in a larger patient population. A larger cohort would enable the models to accurately learn the relationships and retain richer information from a wider set of features.

AI involves computer programs,which can execute functions that we associate with human intelligence, such as learning [46]. AI techniques have shown promises in predicting disease outcomes and are increasingly being used in gastroenterology [47]. To understand the utility of AI in predicting CCA development in PSC, we implemented two AI algorithms, RSF and GBSA, and compared them with the classical CoxPH model. CoxPH relies on the proportional hazards assumptions, whereas RSF and GBSA are free of such assumptions, and hence have the power to uncover complex relationships between predictors and outcomes. However, when sample size is small, RSF and GBSA are prone to overfit the random fluctuations in the training data, leading to suboptimal generalizability on the test set. This is indeed the case in our analysis, especially for GBSA. CoxPH and RSF had the best performance when predicting CCA with clinical variables and laboratory parameters in the baseline cohort. Even though we consciously regularized the RSF and GBSA models through our hyperparameter choices, we still observed a large gap between their training and testing performance, indicating the presence of overfitting. In the BA cohort, using clinical variables and laboratory parameters only, CoxPH again had the best performance, suggesting the proportional hazards assumption may be well suited to capture the relationship between clinical variables, laboratory parameters, and CCA. However, under this assumption, potential nonlinear effects and interactions of the predictors will be ignored. This may be why RSF had better performance than CoxPH when predicting CCA using BAs as predictors. Overfitting in RSF and GBSA was also observed in the BA cohort, suggesting that a larger BA cohort is needed to mitigate overfitting, which may help realize the powerful capacity of the AI algorithms.

Our study has limitations. Although it provides valuable preliminary results on predicting CCA in patients with PSC and showed that individualized predictive models were significantly better than the commonly used risk scores, models with better performance are needed for direct clinical utility. We believe the present work sets the stage for future efforts aimed at development of more accurate CCA risk determination tools, which are desperately needed in clinical practice. The use of cross-sectional data limited our ability to comment on the importance of different risk factors over time, this could be addressed with longitudinal data. The patients in this study were largely seen in academic tertiary high-volume medical centers, and thus are more likely to be inherently complex cases. Furthermore, the frequency of CCA cases among our PSC population is higher compared to other medical centers due to the fact that our institution is a referral center for these diseases. Consequently, the results of our study may not necessarily apply to the entire PSC population. It should be noted that the clinical applicability of our results requires cross-validation in an independent PSC cohort. The criteria we use for CCA diagnosis may be different from other medical centers. The diagnosis of CCA can be extremely challenging especially in patients with PSC due to its silent clinical presentation and lack of accurate and sensitive markers. It requires high clinical suspicion combined with comprehensive laboratory, imaging, and endoscopic evaluation. Positive cytology, although 100% specific for CCA, has very low sensitivity, as low as 20% [48]. Thus, given the limitations of conventional cytology, other cytology techniques have been recently developed. For example, Mayo Clinic investigators have developed and use biliary FISH (fluoresence in situ hybridization) as an additional tool for diagnosis of CCA, and reported a 65% sensitivity for detecting CCA without compromise to specificity [15]. In one study, biliary FISH polysomy was observed in 77% of CCA cases [49]. At our medical center, we use the combination of a malignant stricture and biliary FISH polysomy as criteria for the diagnosis of CCA. In our study, the diagnosis of CCA in a few cases was established based on a malignant appearing stricture and persistent rise in serum CA 19–9 not explained by bacterial cholangitis. Thus, there is a possibility that a small number of patients might not had CCA. The serum IgG4 levels were available for only one quarter of the patients included. However, we note that no PSC patient in the current study cohort had clinical or imaging features to suggest IgG4-related disease (IgG4-RD), and therefore only a very small number of patients could possibly have IgG4-related sclerosing cholangitis (IgG4-SC) and misclassified as having PSC. Different phenotypes of CCA (for example by location, i.e., intrahepatic- and extrahepatic CCA) were combined in our analysis; models based on different CCA phenotypes should be considered in future studies. While we presented plasma BA signatures of CCA in PSC and showed promises for improving CCA prediction, a larger cohort is needed to validate our results. Moreover, imaging techniques such as magnetic resonance imaging/magnetic resonance cholangiopancreatography (MRI/MRCP) provide detailed images of bile ducts and surrounding tissue, and their use has been shown to predict with good accuracy PSC-related complications, such as time to hepatic decompensation and liver-related death, [50–53]. However, data on using MRI/MRCP to predict CCA in patients with PSC are lacking. Adapting our approach to incorporate imaging data might lead to better prediction models in the future. Finally, the complexity of PSC and CCA in PSC requires comprehensive examination and integration of genetic and environmental factors to elucidate the pathophysiology and improve the prediction models. This study is a first step towards a multi-omics based model for individualized CCA prediction in PSC.

## Conclusions

In a large well-documented PSC cohort, we identified clinical and laboratory risk factors for CCA development and examined a statistical learning method and two AI methods that predicted CCA occurrence significantly better than common risk scores. We explored the use of BAs as novel biomarekers, which showed promise for improving CCA prediction. Larger studies and novel biomarker studies are needed for clinical adoption of these models to improve the care of these patients.

## Supplementary Information

**Additional file 1: Fig. S1. **Derivation of baseline cohort and bile acid cohort. Baseline cohort includes 1,459 patients who had pre-outcome laboratory parameters following PSC diagnosis, out of which 118 developed CCA prior to any other outcomes. Bile acid cohort includes 300 patients with pre-outcome plasma bile acids and laboratory parameters collected at similar times, out of which21 developed CCA prior to any other outcomes. Abbreviations: ALK, alkaline phosphatase; AST, alanine aminotransferase; AST, aspartate aminotransferase; CA19-9, carbohydrate antigen 19-9; CCA, cholangiocarcinoma; GBC, gallbladder cancer; IBD, inflammatory bowel disease; HCC, hepatocellular carcinoma; IgG,immunoglobulin; IgG, immunoglobulin G4; INR, international normalized ratio;LT, liver transplantation; MELD, model for end-stage liver disease; PSC,primary sclerosing cholangitis; PREsTo, PSC Risk Estimation Tool. Please see separate list of abbreviations for bile acids. **Fig. S2. **Cumulative incidence function (CIF) of CCA based on a competing risks framework with GBC,HCC, LT and non-PSC death as competing risks. Patients without any events were censored at the last known clinical encounter. CIF of CCA represents the cumulative probability of developing CCA (without developing any other events).Abbreviations: CCA, cholangiocarcinoma; GBC, gallbladder cancer; HCC,hepatocellular carcinoma; LT, liver transplantation; PSC, primary sclerosing cholangitis. **Table S1. **Summary characteristics of the bile acid cohort with median (IQR) listed for the continuous features and percentage listed for the binary features.

## Data Availability

The datasets used and/or analyzed during the current study are available from the corresponding author on reasonable request.

## Abbreviations

PSC: Primary sclerosing cholangitis
LT: Liver transplantation
IBD: Inflammatory bowel disease
CCA: Cholangiocarcinoma
CA 19-9: Carbohydrate antigen 19–9
BA: Bile acid
PREsTo: Primary sclerosing cholangitis Risk Estimate Tool
AI: Artificial intelligence
FISH: Fluorescence in situ hybridization
GBC: Gallbladder cancer
HCC: Hepatocellular carcinoma
EMR: Electronic medical record
MELD: Model for End-stage Liver Disease
ALK: Alkaline phosphatase
ALT: Alanine aminotransferase
AST: Aspartate aminotransferase
INR: International normalized ratio
MICE: Multivariate imputation by chained equations
CA: Cholic acid
CDCA: Chenodeoxycholic acid
DCA: Deoxycholic acid
LCA: Lithocholic acid
UDCA: Ursodeoxycholic acid
HDCA: Hyodeoxycholic acid
TCA: Taurocholic acid
TCDCA: Taurochenodeoxycholic acid
TDCA: Taurodeoxycholic acid
TLCA: Taurolithocholic acid
TUDCA: Tauroursodeoxycholic acid
THDCA: Taurohyodeoxycholic acid
GCA: Glycocholic acid
GCDCA: Glycochenodeoxycholic acid
GDCA: Glycodeoxycholic acid
GLCA: Glycolithocholic acid
GUDCA: Glycoursodeoxycholic acid
GHDCA: Glycohyodeoxycholic acid
CIF: Cumulative incidence function
CoxPH: Cox proportional hazards model
HR: Hazard ratio
IQR: Interquartile range
CI: Confidence interval
FDR: False discovery rate
RSF: Random survival forest
GBSA: Gradient boosting survival analysis
C-index: Concordance index
