# Supplementary material for: Predicting cholangiocarcinoma in primary sclerosing cholangitis: using artificial intelligence, clinical and laboratory data
Source: BMC Gastroenterol. 2023 Apr 19;23:129. doi: 10.1186/s12876-023-02759-7 (PMC10114387; doi:10.1186/s12876-023-02759-7)
Supplement: Supplementary file 1 — Additional file 1: Fig. S1. Derivation of baseline cohort and bile acid cohort. Baseline cohort includes 1,459 patients who had pre-outcome laboratory parameters following PSC diagnosis, out of which 118 developed CCA prior to any other outcomes. Bile acid cohort includes 300 patients with pre-outcome plasma bile acids and laboratory parameters collected at similar times, out of which21 developed CCA prior to any other outcomes. Abbreviations: ALK, alkaline phosphatase; AST, alanine aminotransferase; AST, aspartate aminotransferase; CA19-9, carbohydrate antigen 19-9; CCA, cholangiocarcinoma; GBC, gallbladder cancer; IBD, inflammatory bowel disease; HCC, hepatocellular carcinoma; IgG,immunoglobulin; IgG, immunoglobulin G4; INR, international normalized ratio;LT, liver transplantation; MELD, model for end-stage liver disease; PSC,primary sclerosing cholangitis; PREsTo, PSC Risk Estimation Tool. Please see separate list of abbreviations for bile acids. Fig. S2. Cumulative incidence function (CIF) of CCA based on a competing risks framework with GBC,HCC, LT and non-PSC death as competing risks. Patients without any events were censored at the last known clinical encounter. CIF of CCA represents the cumulative probability of developing CCA (without developing any other events).Abbreviations: CCA, cholangiocarcinoma; GBC, gallbladder cancer; HCC,hepatocellular carcinoma; LT, liver transplantation; PSC, primary sclerosing cholangitis. Table S1. Summary characteristics of the bile acid cohort with median (IQR) listed for the continuous features and percentage listed for the binary features. [file 12876_2023_2759_MOESM1_ESM.docx]

**Supplemental Figures**

**Fig. S1** Derivation of baseline cohort and bile acid cohort. Baseline cohort includes 1,459 patients who had pre-outcome laboratory parameters following PSC diagnosis, out of which 118 developed CCA prior to any other outcomes. Bile acid cohort includes 300 patients with pre-outcome plasma bile acids and laboratory parameters collected at similar times, out of which 21 developed CCA prior to any other outcomes. Abbreviations: ALK, alkaline phosphatase; AST, alanine aminotransferase; AST, aspartate aminotransferase; CA 19-9, carbohydrate antigen 19-9; CCA, cholangiocarcinoma; GBC, gallbladder cancer; IBD, inflammatory bowel disease; HCC, hepatocellular carcinoma; IgG, immunoglobulin; IgG, immunoglobulin G4; INR, international normalized ratio; LT, liver transplantation; MELD, model for end-stage liver disease; PSC, primary sclerosing cholangitis; PREsTo, PSC Risk Estimation Tool. Please see separate list of abbreviations for bile acids.

**Fig. S2** Cumulative incidence function (CIF) of CCA based on a competing risks framework with GBC, HCC, LT and non-PSC death as competing risks. Patients without any events were censored at the last known clinical encounter. CIF of CCA represents the cumulative probability of developing CCA (without developing any other events). Abbreviations: CCA, cholangiocarcinoma; GBC, gallbladder cancer; HCC, hepatocellular carcinoma; LT, liver transplantation; PSC, primary sclerosing cholangitis.

**Supplemental Table**

**Table S1** Summary characteristics of the bile acid cohort with median (IQR) listed for the continuous features and percentage listed for the binary features.

| **Characteristics of Bile Acid Cohort** | | |
| --- | --- | --- |
|  | | |
| Variable | N | Summary |
|  | | |
| Age, years | 300 | 49.9 (35.16–61.16) |
| Male sex | 300 | 58.0% |
| PSC duration, years | 300 | 4.16 (1.85–9.47) |
| IBD diagnosis | 300 | 76.7% |
| IBD duration, years | 230 | 13.76 (4.86–24.59) |
| ALK x ULN | 290 | 1.57 (0.89–2.87) |
| AST, U/L | 286 | 45 (30–79.5) |
| ALT, U/L | 254 | 55 (32–111) |
| Total bilirubin, mg/dL | 286 | 0.8 (0.5–1.2) |
| Direct bilirubin, mg/dL | 194 | 0.2 (0.1–0.4) |
| INR | 246 | 1 (1–1.1) |
| Albumin, g/dL | 274 | 4.15 (3.8–4.4) |
| Sodium, mmol/L | 168 | 139.5 (138–142) |
| Creatinine, mg/dL | 243 | 0.9 (0.8–1) |
| Hemoglobin, g/dL | 218 | 14.1 (12.9–15) |
| White Blood Cells, 10^9^/L | 203 | 6.2 (5–7.5) |
| Platelets, 10^9^/L | 271 | 222 (173.5–279) |
| IgG4, mg/dL | 66 | 32 (17.98–73.8) |
| Total IgG, mg/dL | 62 | 1315 (1070–1737.5) |
| CA 19-9, U/mL | 225 | 17 (7.2–32) |
| LCA, μM | 300 | 0.01 (0–0.04) |
| CDCA, μM | 300 | 0.15 (0.05–0.43) |
| DCA, μM | 300 | 0.09 (0.01–0.45) |
| UDCA, μM | 300 | 0.15 (0.01–1.73) |
| HDCA, μM | 300 | 0.01 (0–0.03) |
| CA, μM | 300 | 0.07 (0.03–0.23) |
| GLCA, μM | 300 | 0.02 (0–0.08) |
| GCDCA, μM | 300 | 3 (1.1–6.9) |
| GDCA, μM | 300 | 0.32 (0.03–0.98) |
| GUDCA, μM | 300 | 0.96 (0.12–12.06) |
| GHDCA, μM | 300 | 0 (0–0.01) |
| GCA, μM | 300 | 1.75 (0.46–6.45) |
| TLCA, μM | 300 | 0 (0–0.02) |
| TCDCA, μM | 300 | 0.52 (0.13–2.3) |
| TDCA, μM | 300 | 0.06 (0.01–0.26) |
| TUDCA, μM | 300 | 0.08 (0.01–0.44) |
| THDCA, μM | 300 | 0 (0–0) |
| TCA, μM | 300 | 0.26 (0.06–2.53) |
| Total Bile Acids, μM | 300 | 17.12 (5.91–41.38) |
| Total CA, μM | 300 | 2.17 (0.8–10.01) |
| Total CDCA, μM | 300 | 4.1 (1.58–10.2) |
| Total DCA, μM | 300 | 0.66 (0.06–1.78) |
| Total LCA, μM | 300 | 0.04 (0–0.15) |
| Total UDCA, μM | 300 | 1.32 (0.19–15.05) |
| Total HDCA, μM | 300 | 0.01 (0–0.04) |
| ConFrac CA | 300 | 0.97 (0.84–0.99) |
| ConFrac CDCA | 300 | 0.96 (0.84–0.99) |
| ConFrac DCA | 261 | 0.8 (0.55–0.96) |
| ConFrac LCA | 217 | 0.75 (0.55–0.87) |
| ConFrac UDCA | 294 | 0.89 (0.75–0.97) |
| ConFrac HDCA | 178 | 0.18 (0–0.55) |
| ConFrac all BA | 300 | 0.91 (0.74–0.98) |
| GTratio CA | 295 | 5.56 (2.59–9.37) |
| GTratio CDCA | 299 | 5.86 (2.75–10.3) |
| GTratio DCA | 223 | 4.69 (2.07–8.96) |
| GTratio LCA | 130 | 3 (1.34–7) |
| GTratio UDCA | 247 | 19.23 (6.46–36.03) |
| GTratio HDCA | 10 | 1.17 (1–2.6) |
| GTratio all BA | 299 | 8.49 (3.2–17.44) |
| CA:CDCA | 300 | 0.66 (0.36–1.13) |
| CA:DCA | 261 | 2.57 (0.81–28.14) |
| CDCA:(LCA+HDCA+UDCA) | 294 | 2.27 (0.22–16.46) |
|  |  |  |
